# The genome sequence of the long-horned flat-body, *Carcina quercana *(Fabricius, 1775)

**DOI:** 10.12688/wellcomeopenres.18596.1

**Published:** 2023-01-12

**Authors:** Douglas Boyes, University of Oxford and Wytham Woods Genome Acquisition Lab, Darwin Tree of Life Barcoding collective, Wellcome Sanger Institute Tree of Life programme, Wellcome Sanger Institute Scientific Operations: DNA Pipelines collective, Tree of Life Core Informatics collective, David Lees, Darwin Tree of Life Consortium

**Affiliations:** 1 UK Centre for Ecology and Hydrology, Wallingford, Oxfordshire, UK; 2 Natural History Museum, London, UK

**Keywords:** Carcina quercana, long-horned flat-body, genome sequence, chromosomal, Lepidoptera

## Abstract

We present a genome assembly from an individual male *Carcina quercana *(the long-horned flat-body; Arthropoda; Insecta; Lepidoptera; Depressariidae). The genome sequence is 409 megabases in span. Most of the assembly (99.96%) is scaffolded into 30 chromosomal pseudomolecules, including the assembled Z sex chromosome. The complete mitochondrial genome was also assembled and is 15.3 kilobases in length. Gene annotation of this assembly on Ensembl identified 18,108 protein coding genes.

## Species taxonomy

Eukaryota; Metazoa; Ecdysozoa; Arthropoda; Hexapoda; Insecta; Pterygota; Neoptera; Endopterygota; Lepidoptera; Glossata; Ditrysia; Gelechioidea; Depressariidae; Depressariidae incertae sedis; *Carcina*; *Carcina quercana* (Fabricius, 1775) (NCBI:txid116121).

## Background

The long-horned flat-body, *Carcina quercana* (Fabricius, 1775), is a micromoth belonging to the Depressariidae family. It can be identified by its pastel purple and yellow wing patterning and notably long antennae. In the Western Palaearctic, *C. quercana* is widespread in Europe, including the UK, and reaches its eastern limit in the Middle East. The species has also recently been introduced into North America. Across its range, *C. quercana is* generally common but rarely abundant.

The species prefers woodland and garden habitats and is moderately polyphagous on deciduous trees, favouring species within the Fagaceae family ( *Quercus* and *Fagus* spp.) and the Rosaceae family. Adults fly from May to October, peaking in July, and produce larvae that skeletonise under a silken web. *C. quercana* has been described as a minor pest of Rosaceae fruit trees, such as apple, pear, cherry and plum among others ( Alford, 2016).

*Carcina quercana* represents a lineage otherwise not present in Europe and is thus of phylogenomic value. It is the only UK representative of the Peleopodidae (hitherto usually included as a subfamily of Depressariidae). This gelechioid family turns out to have previously unsuspected richness in the Old World tropics, containing various lineages previously placed in Oecophoridae and Depressariidae ( Wang & Li, 2020), but the species has not been included generally in multi-genomic studies to date.

### Genome sequence report

**Figure 1.**
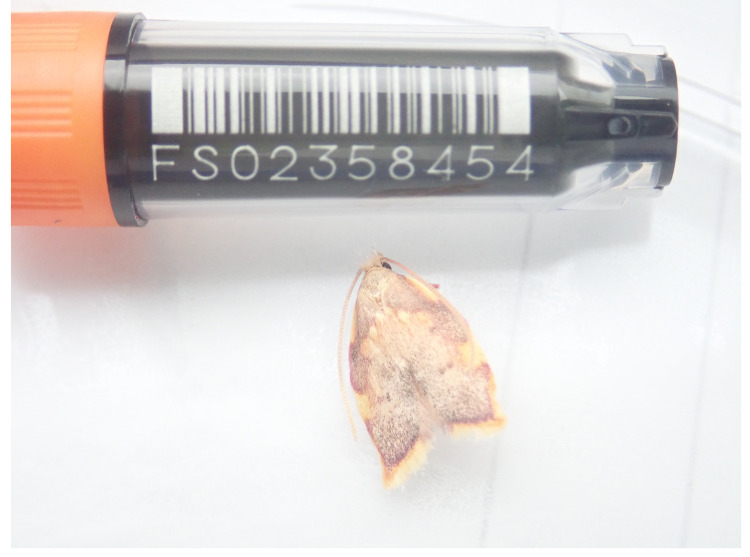
Image of the *Carcina quercana* specimen taken prior to preservation and processing.

The genome was sequenced from a single male *C. quercana* ( Figure 1) collected from Ant Hills region, Wytham, Berkshire, UK. A total of 57-fold coverage in Pacific Biosciences single-molecule HiFi long reads and 99-fold coverage in 10X Genomics read clouds were generated. Primary assembly contigs were scaffolded with chromosome conformation Hi-C data. Manual assembly curation corrected 15 missing/misjoins, reducing the assembly size by 0.23% and the scaffold number by 32.61%, and increasing the scaffold N50 by 10.28%.

**Figure 2.**
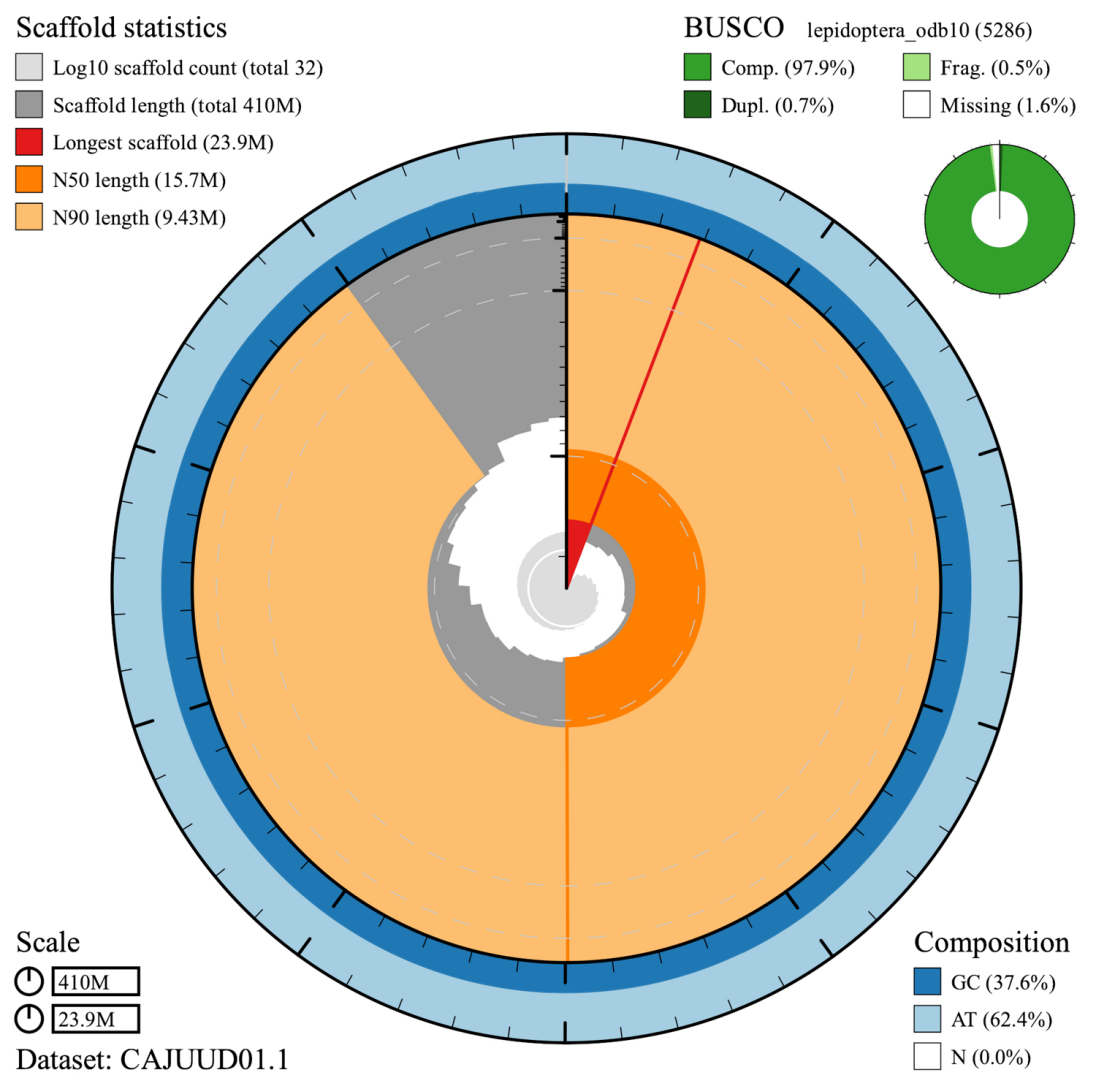
Genome assembly of *Carcina quercana*, ilCarQuer1.1: metrics. The BlobToolKit Snailplot shows N50 metrics and BUSCO gene completeness. The main plot is divided into 1,000 size-ordered bins around the circumference with each bin representing 0.1% of the 409,517,697 bp assembly. The distribution of chromosome lengths is shown in dark grey with the plot radius scaled to the longest chromosome present in the assembly (23,876,981 bp, shown in red). Orange and pale-orange arcs show the N50 and N90 chromosome lengths (15,733,321 and 9,427,983 bp), respectively. The pale grey spiral shows the cumulative chromosome count on a log scale with white scale lines showing successive orders of magnitude. The blue and pale-blue area around the outside of the plot shows the distribution of GC, AT and N percentages in the same bins as the inner plot. A summary of complete, fragmented, duplicated and missing BUSCO genes in the lepidoptera_odb10 set is shown in the top right. An interactive version of this figure is available at https://blobtoolkit.genomehubs.org/view/ilCarQuer1.1/dataset/CAJUUD01.1/snail.

The final assembly has a total length of 409 Mb in 31 sequence scaffolds with a scaffold N50 of 15.7 Mb ( Table 1). Most of the assembly sequence (99.96%) was assigned to 30 chromosomal-level scaffolds, representing 29 autosomes (numbered by sequence length) and the Z sex chromosome ( Figure 2– Figure 5; Table 2).

**Table 1.** Genome data for *Carcina quercana*, ilCarQuer1.2.

*Project accession data*	
Assembly identifier	ilCarQuer1.2
Species	*Carcina quercana*
Specimen	ilCarQuer1 (genome assembly); ilCarQuer2 (Hi-C)
NCBI taxonomy ID	116121
BioProject	PRJEB45132
BioSample ID	SAMEA7519850
Isolate information	Male, whole organism (ilCarQuer1); whole organism (ilCarQuer2)
*Raw data accessions*	
PacificBiosciences SEQUEL II	ERR6394586; ERR6558186
10X Genomics Illumina	ERR6054827–ERR6054830
Hi-C Illumina	ERR6054831
*Genome assembly*	
Assembly accession	GCA_910589575.2
*Accession of alternate haplotype*	GCA_910589345.2
Span (Mb)	409
Number of contigs	56
Contig N50 length (Mb)	13.0
Number of scaffolds	31
Scaffold N50 length (Mb)	15.7
Longest scaffold (Mb)	23.9
BUSCO * genome score	C:97.9%[S:97.2%,D:0.7%], F:0.5%,M:1.6%,n:5,286
*Genome annotation*	
Number of protein-coding genes	18,108

*BUSCO scores based on the lepidoptera_odb10 BUSCO set using v5.3.2. C = complete [S = single copy, D = duplicated], F = fragmented, M = missing, n = number of orthologues in comparison. A full set of BUSCO scores is available at https://blobtoolkit.genomehubs.org/view/ilCarQuer1.1/dataset/CAJUUD01.1/busco.

**Figure 3.**
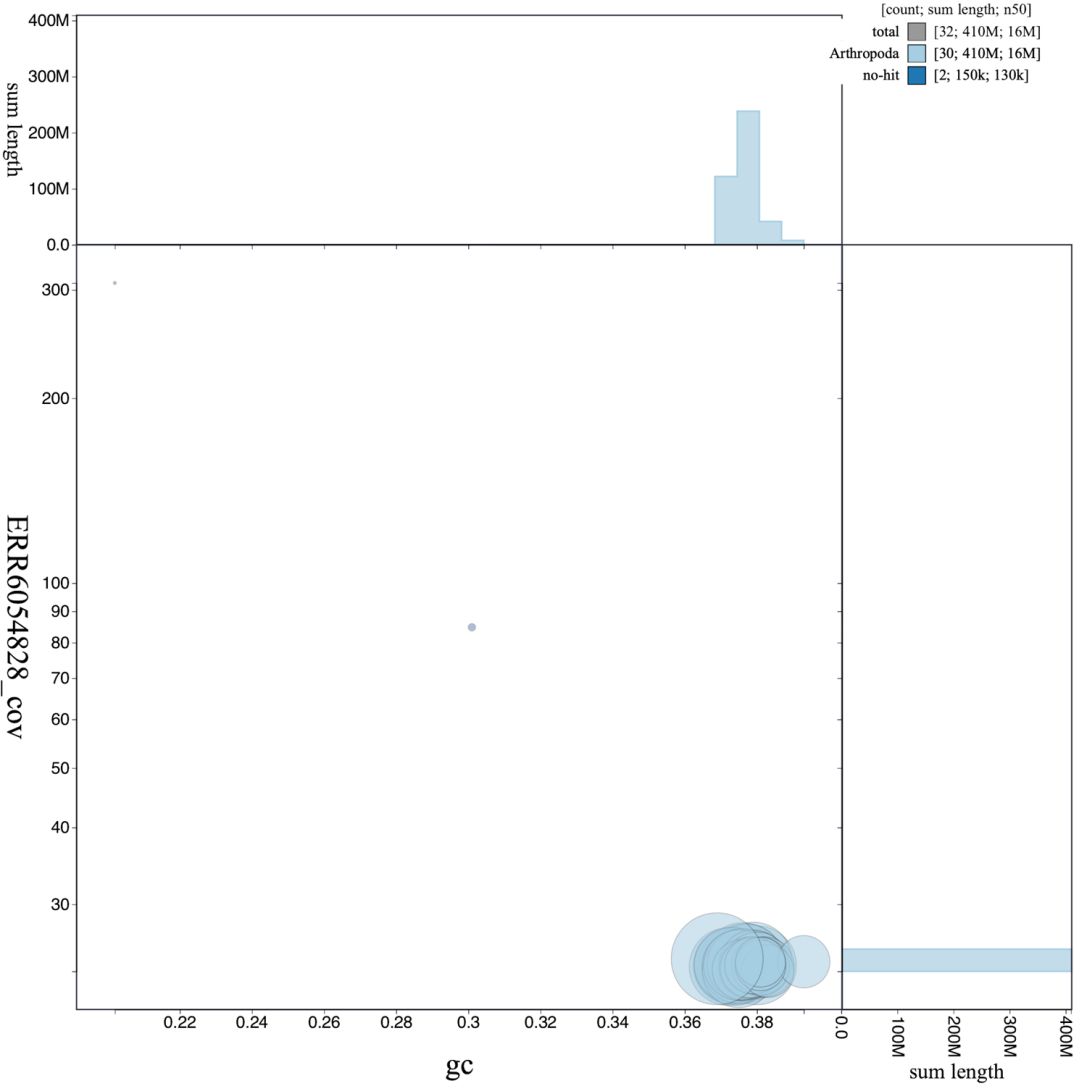
Genome assembly of *Carcina quercana*, ilCarQuer1.1: GC coverage. BlobToolKit GC-coverage plot. Scaffolds are coloured by phylum. Circles are sized in proportion to scaffold length. Histograms show the distribution of scaffold length sum along each axis. An interactive version of this figure is available at https://blobtoolkit.genomehubs.org/view/ilCarQuer1.1/dataset/CAJUUD01.1/blob.

**Figure 4.**
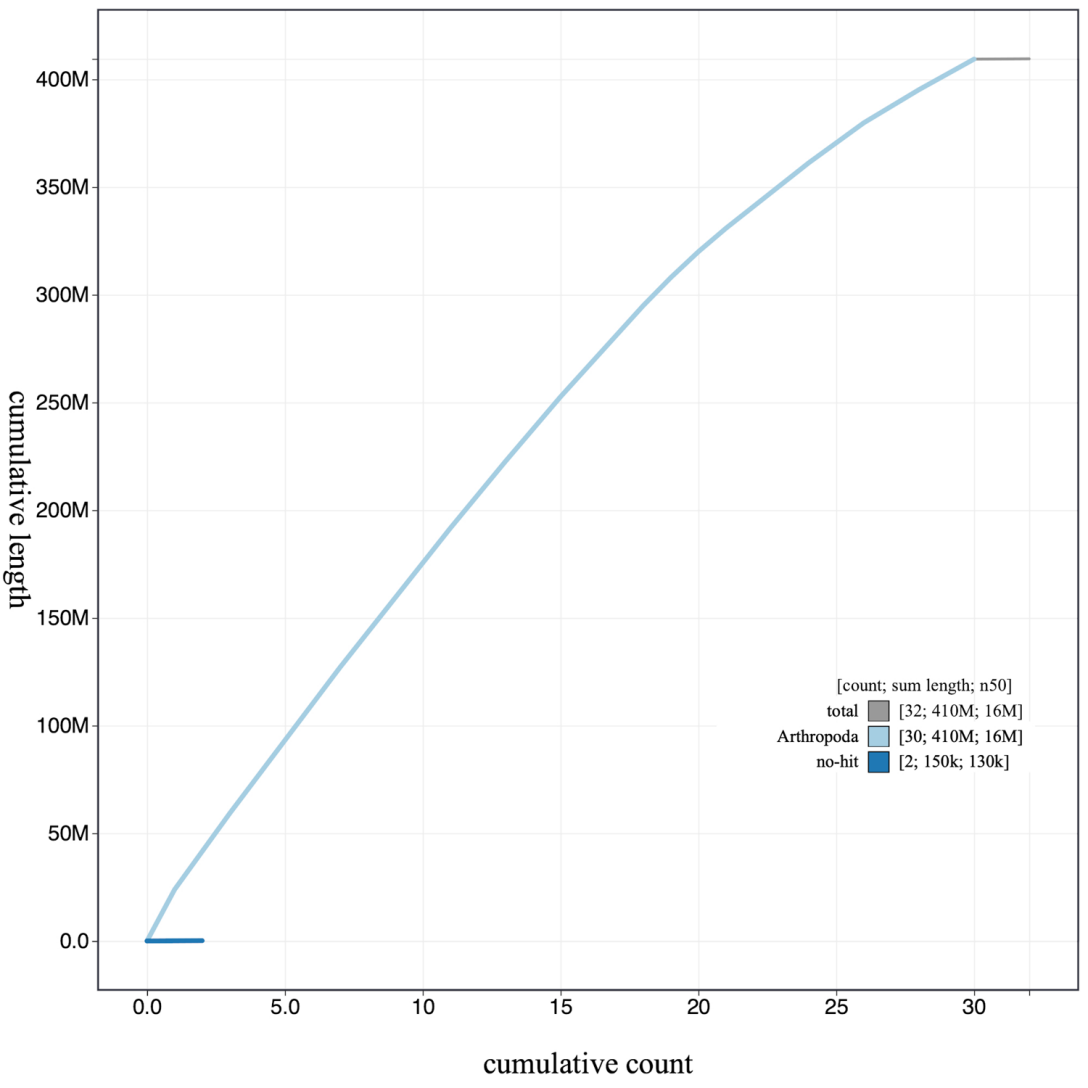
Genome assembly of *Carcina quercana*, ilCarQuer1.1: cumulative sequence. BlobToolKit cumulative sequence plot. The grey line shows cumulative length for all scaffolds. Coloured lines show cumulative lengths of scaffolds assigned to each phylum using the buscogenes taxrule. An interactive version of this figure is available at https://blobtoolkit.genomehubs.org/view/ilCarQuer1.1/dataset/CAJUUD01.1/cumulative.

**Figure 5.**
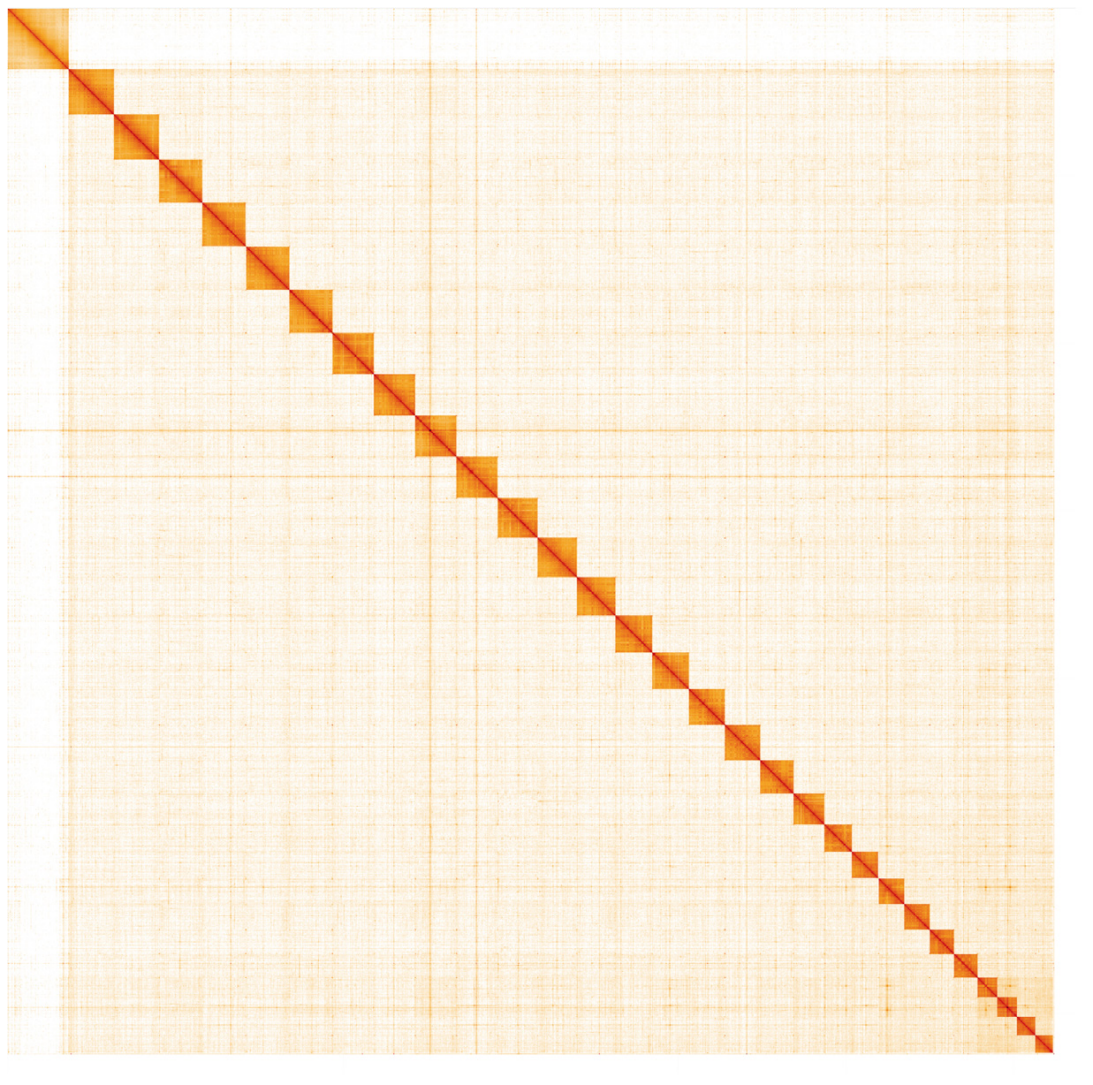
Genome assembly of *Carcina quercana*, ilCarQuer1.1: Hi-C contact map. Hi-C contact map of the ilCarQuer1.1 assembly, visualised in HiGlass. Chromosomes are arranged in size order from left to right and top to bottom. The interactive Hi-C map can be viewed at https://genome-note-higlass.tol.sanger.ac.uk/l/?d=VCvcSg41Qm2RTmIMdU-0pA.

**Table 2.** Chromosomal pseudomolecules in the genome assembly of *Carcina quercana*, ilCarQuer1.2.

INSDC accession	Chromosome	Size (Mb)	GC%
OU342427.1	1	17.8	37.6
OU342428.1	2	17.56	38
OU342429.1	3	17.05	37.6
OU342430.1	4	16.95	37.2
OU342431.1	5	16.93	37.4
OU342432.1	6	16.84	37.9
OU342433.1	7	16.2	37.4
OU342434.1	8	16.2	37.3
OU342435.1	9	16.19	37.7
OU342436.1	10	16.02	37.3
OU342437.1	11	15.73	37.3
OU342438.1	12	15.27	37.5
OU342439.1	13	15.14	37.5
OU342440.2	14	14.26	37.7
OU342441.1	15	14.27	37.6
OU342442.1	16	14.04	37.6
OU342443.1	17	14	37.5
OU342444.1	18	13.03	37.9
OU342445.1	19	11.99	38
OU342446.1	20	10.89	37.8
OU342447.1	21	10.26	37.6
OU342448.1	22	10.18	38.2
OU342449.1	23	9.91	37.8
OU342450.1	24	9.43	38.1
OU342451.1	25	9.17	37.9
OU342452.1	26	7.71	38.3
OU342453.1	27	7.66	39.3
OU342454.1	28	7.21	38.1
OU342455.1	29	6.99	38.1
OU342426.1	Z	23.88	36.9
OU342456.1	MT	0.02	20.5

The assembly has a BUSCO v5.3.2 ( Manni *et al.*, 2021) completeness of 97.9% (single 97.2%, duplicated 0.7%) using the lepidoptera_odb10 reference set ( *n* = 5,286). While not fully phased, the assembly deposited is of one haplotype. Contigs corresponding to the second haplotype have also been deposited.

### Genome annotation report

The ilCarQuer1.1 genome was annotated using BRAKER2 ( Brůna *et al.*, 2021) ( Table 1; Ensembl annotation). The resulting annotation includes 18,272 transcribed mRNAs from 18,108 protein-coding genes.

## Methods

### Sample acquisition and nucleic acid extraction

A single male *C. quercana* specimen (ilCarQuer1) was collected using a light trap from Ant Hills region, Wytham, Berkshire, UK (latitude 51.765, longitude –1.327) by Douglas Boyes (University of Oxford). A second *C. quercana* specimen (ilCarQuer2) (unsexed individual) was collected using a light trap from Wytham Woods, Berkshire, UK (latitude 51.765, longitude –1.335) by Douglas Boyes (University of Oxford). Both specimens were identified by Douglas Boyes and snap-frozen on dry ice.

DNA was extracted at the Scientific Operations Core, Wellcome Sanger Institute. The ilCarQuer1 sample was weighed and dissected on dry ice. Whole organism tissue was disrupted by manual grinding in a lysis buffer with a disposable pestle. Fragment size analysis of 0.01–0.5 ng of DNA was then performed using an Agilent FemtoPulse. High molecular weight (HMW) DNA was extracted using the Qiagen MagAttract HMW DNA extraction kit. Low molecular weight DNA was removed from a 200 ng aliquot of extracted DNA using 0.8X AMpure XP purification kit prior to 10X Chromium sequencing, and a minimum of 50 ng DNA was submitted for 10X sequencing. HMW DNA was sheared into an average fragment size of 12–20 kb in a Megaruptor 3 system with speed setting 30. Sheared DNA was purified by solid-phase reversible immobilisation using AMPure PB beads with a 1.8X ratio of beads to sample to remove the shorter fragments and concentrate the DNA sample. The concentration of the sheared and purified DNA was assessed using a Nanodrop spectrophotometer and Qubit Fluorometer and Qubit dsDNA High Sensitivity Assay kit. Fragment size distribution was evaluated by running the sample on the FemtoPulse system.

### Sequencing

Pacific Biosciences HiFi circular consensus and 10X Genomics Chromium read cloud sequencing libraries were constructed according to the manufacturers’ instructions. Sequencing was performed by the Scientific Operations core at the Wellcome Sanger Institute on Pacific Biosciences SEQUEL II (HiFi) and Illumina HiSeq (10X) instruments. Hi-C data were generated in the Tree of Life laboratory from whole organism tissue of ilCarQuer2 using the Arima v2 kit and sequenced on a NovaSeq 6000 instrument. 

### Genome assembly

Assembly was carried out with Hifiasm ( Cheng *et al.*, 2021) and haplotypic duplication was identified and removed with purge_dups ( Guan *et al.*, 2020). One round of polishing was performed by aligning 10X Genomics read data to the assembly with longranger align, calling variants with freebayes ( Garrison & Marth, 2012). The assembly was then scaffolded with Hi-C data ( Rao *et al.*, 2014) using SALSA2 ( Ghurye *et al.*, 2019). The assembly was checked for contamination and corrected using the gEVAL system ( Chow *et al.*, 2016) as described previously ( Howe *et al.*, 2021). Manual curation ( Howe *et al.*, 2021) was performed using gEVAL, HiGlass ( Kerpedjiev *et al.*, 2018) and Pretext ( Harry, 2022). The mitochondrial genome was assembled using MitoHiFi ( Uliano-Silva *et al.*, 2022), which performs annotation using MitoFinder ( Allio *et al.*, 2020). The genome was analysed and BUSCO scores were generated within the BlobToolKit environment ( Challis *et al.*, 2020). Table 3 contains a list of all software tool versions used, where appropriate.

**Table 3.** Software tools used.

Software tool	Version	Source
Hifiasm	0.15.1	( Cheng *et al.*, 2021)
purge_dups	1.2.3	( Guan *et al.*, 2020)
SALSA2	2.2	( Ghurye *et al.*, 2019)
longranger align	2.2.2	https://support.10xgenomics.com/genome-exome/ software/pipelines/latest/advanced/other-pipelines
freebayes	1.3.1-17- gaa2ace8	( Garrison & Marth, 2012)
MitoHiFi	2.0	( Uliano-Silva *et al.*, 2022)
gEVAL	N/A	( Chow *et al.*, 2016)
HiGlass	1.11.6	( Kerpedjiev *et al.*, 2018)
PretextView	0.2.x	https://github.com/wtsi-hpag/PretextView
BlobToolKit	3.2.6	( Challis *et al.*, 2020)

### Genome annotation

The BRAKER2 gene annotation system ( Brůna *et al.*, 2021) was used to generate annotation for the *C. quercana* ilCarQuer1.2 assembly (GCA_910589575.1).

### Ethics/compliance issues

The materials that have contributed to this genome note have been supplied by a Darwin Tree of Life Partner. The submission of materials by a Darwin Tree of Life Partner is subject to the Darwin Tree of Life Project Sampling Code of Practice. By agreeing with and signing up to the Sampling Code of Practice, the Darwin Tree of Life Partner agrees they will meet the legal and ethical requirements and standards set out within this document in respect of all samples acquired for, and supplied to, the Darwin Tree of Life Project. Each transfer of samples is further undertaken according to a Research Collaboration Agreement or Material Transfer Agreement entered into by the Darwin Tree of Life Partner, Genome Research Limited (operating as the Wellcome Sanger Institute), and in some circumstances other Darwin Tree of Life collaborators.

## Data Availability

European Nucleotide Archive: Carcina quercana (long-horned flat-body). Accession number PRJEB45132; https://identifiers.org/ena.embl/PRJEB45132 ( Wellcome Sanger Institute, 2022). The genome sequence is released openly for reuse. The *C. quercana* genome sequencing initiative is part of the Darwin Tree of Life (DToL) project. All raw sequence data and the assembly have been deposited in INSDC databases. Raw data and assembly accession identifiers are reported in Table 1.

## Author information

Members of the University of Oxford and Wytham Woods Genome Acquisition Lab are listed here: https://doi.org/10.5281/zenodo.4789928.

Members of the Darwin Tree of Life Barcoding collective are listed here: https://doi.org/10.5281/zenodo.4893703.

Members of the Wellcome Sanger Institute Tree of Life programme are listed here: https://doi.org/10.5281/zenodo.4783585.

Members of Wellcome Sanger Institute Scientific Operations: DNA Pipelines collective are listed here: https://doi.org/10.5281/zenodo.4790455.

Members of the Tree of Life Core Informatics collective are listed here: https://doi.org/10.5281/zenodo.5013541.

Members of the Darwin Tree of Life Consortium are listed here: https://doi.org/10.5281/zenodo.4783558.
