# Supplementary material for: The genome sequence of the long-horned flat-body, Carcina quercana (Fabricius, 1775)
Source: Wellcome Open Res. 2023 Jan 12;8:16. [Version 1] doi: 10.12688/wellcomeopenres.18596.1 (PMC10220420; doi:10.12688/wellcomeopenres.18596.1)
Supplement: Peer review history

# Peer review: Reviewer response for version 1

**DOI:** 10.21956/wellcomeopenres.20621.r58381

**Published:** 2023-05-26

**Authors:** Robert M Waterhouse

**Affiliations:** 1 Department of Ecology and Evolution & Swiss Institute of Bioinformatics, University of Lausanne, Lausanne, Switzerland

This Data Note for the long-horned flat-body micromoth presents a clear and comprehensive description of all the steps taken to generate the *Carcina quercana *genome assembly with 31 scaffolds spanning 409 Mbp with >99% assigned to 30 chromosomal-level scaffolds including the Z sex chromosome, and to produce protein-coding gene annotations via Ensembl. Although this micromoth is considered only a minor pest, it does impact apple, pear, cherry and plum fruit trees, more importantly regarding the rationale for building these resources is that this species represents a lineage otherwise not present in Europe. The described data collection and analysis methods follow the best practices in the field and have delivered a high-quality complete and accurate chromosome-level reference genome. The genome assembly is of one haplotype, with contigs corresponding to the second haplotype also deposited. Gene annotations for 18,108 protein-coding genes are provided with the genome assembly.

Are sufficient details of methods and materials provided to allow replication by others?

Yes

Is the rationale for creating the dataset(s) clearly described?

Yes

Are the datasets clearly presented in a useable and accessible format?

Yes

Are the protocols appropriate and is the work technically sound?

Yes

Reviewer Expertise:

Arthropod evolutionary genomics

I confirm that I have read this submission and believe that I have an appropriate level of expertise to confirm that it is of an acceptable scientific standard.

# Peer review: Reviewer response for version 1

**DOI:** 10.21956/wellcomeopenres.20621.r54063

**Published:** 2023-02-13

**Authors:** Beatriz Vicoso

**Affiliations:** 1 Institute of Science and Technology Austria, Klosterneuburg, Austria

A chromosome level assembly, obtained from PacBio Hifi reads and HiC data for the micromoth *Carcina quercana*, is presented in this manuscript. It consists of 30 pseudo-chromosomes, including the Z-chromosome, that include over 99% of the assembled sequence.  As expected for such a high quality assembly, the BUSCO score is >98%. This therefore represents a great resource for a species for which there were until now no genomic resources. I do not have any criticism, just some very minor suggestions for text edits.

 The rationale for getting the data is well described, as this species is the only representative of the Peleopodidae subfamily in the UK. Some words about what resources are available for this group outside of the UK would have been helpful (I could not find any other than the Illumina sequencing of *Acria ceramitis*, and that seems perhaps worth emphasizing).

 The protocols are appropriate and the methods very well described. The only thing that could have been added would have been information about what parameters were changed from the default for each of the software listed in Table 3 (or specify that default parameters were used throughout).

 A short sentence explaining conceptually how the gene annotation was performed without RNA would also have been helpful.

 The data are easily accessed once one finds the correct page on the Darwin Tree of Life website. A direct link to this page would have been helpful.

Are sufficient details of methods and materials provided to allow replication by others?

Yes

Is the rationale for creating the dataset(s) clearly described?

Yes

Are the datasets clearly presented in a useable and accessible format?

Yes

Are the protocols appropriate and is the work technically sound?

Yes

Reviewer Expertise:

NA

I confirm that I have read this submission and believe that I have an appropriate level of expertise to confirm that it is of an acceptable scientific standard.
